# Co-Circulation and Persistence of Genetically Distinct Saffold Viruses, Denmark

**DOI:** 10.3201/eid1810.120793

**Published:** 2012-10

**Authors:** Alex Christian Yde Nielsen, Mette Louise Gyhrs, Edward C. Holmes, Jie Cui

**Affiliations:** Statens Serum Institut, Copenhagen, Denmark (A.C.Y. Nielsen);; University Hospital of Hobaek, Denmark (M.L. Ghyrs);; University Hospital of Odense, Odense, Denmark (A.C.Y. Nielsen);; Pennsylvania State University, University Park, Pennsylvania, USA (E.C. Holmes, J. Cui);; and National Institutes of Health, Bethesda, Maryland, USA (E.C. Holmes)

**Keywords:** Safford virus, cardiovirus, evolution, phylogeny, co-circulation, persistence, Denmark, viruses, *Suggested citation for this article*: Christian A, Nielsen Y, Gyhrs ML, Holmes EC, Cui J. Co-circulation and persistence of genetically distinct Saffold viruses, Denmark. Emerg Infect Dis [Internet]. 2012 Oct [*date cited*]. http://dx.doi.org/10.3201/eid1810.120793

**To the Editor:** Cardioviruses are positive-sense, single-stranded RNA viruses of the family *Picornaviridae*, genus *Cardiovirus*. Until recently, cardioviruses were primarily known for their ability to infect rodents. In 2007, findings of a retrospective study of undiagnosed enteric illnesses in the United States were published, including results from analysis of a fecal sample from an infant girl whose symptoms were diagnosed as fever of unknown origin in 1981. The novel human cardiovirus that was isolated was designated Saffold virus (SAFV) (*1*). Eight genotypes of SAFV have been described (*1*–*4*), and a ninth was recently isolated in Nigeria (O. Blinkova, unpub. data). Serologic studies indicate that infection with SAFV genotypes 2 and 3 generally occurs in early life (*5*), although the clinical significance of these findings remains unclear.

The first SAFV infection in Denmark was recorded in 2009 (*6*). To elucidate the molecular epidemiology of SAFV, we performed a 3-year surveillance study of SAFV in Denmark. During 2009–2011, we tested 1,393 fecal samples from 454 children. Surveillance included collection of fecal samples from children at 6, 10, and 15 months of age; additional fecal samples were collected when the children had gastroenteritis. Most of the SAFV-positive samples reported in this study were obtained from a randomized trial in the pediatric department of University Hospital of Holbaek (Holbaek) on the effect of probiotic therapy on the incidence of infection during early childhood (M. Gyhrs, unpub. data). The study was approved by the local ethics committee; Den Regionale Videnskabsetiske Komité for Region Sjaelland, Denmark.

Nucleic acids were extracted from 200-µL fecal suspension (10% in phosphate-buffered saline) by using the Cobas AmpliPrep Total Nucleic Acid Isolation Kit (Roche Diagnostics, Ltd., Mannheim, Germany) on the MagnaPure LC instrument (Roche Diagnostics). We used 5 µL of extracted nucleic acid for reverse transcription PCR (RT-PCR) (total volume 25 µL) using the OneStep RT-PCR Kit (QIAGEN, Hilden, Germany). The samples were tested for SAFV by using real-time RT-PCR primer/probe, and all positive samples were genotyped by partial sequencing of the viral protein (VP) 1 gene (*6*). Overall, 38 (2.8%) of the clinical samples were positive for SAFV (Technical Appendix), all of which fell into genotype 2 (SAFV-2), which is most prevalent in Western nations. Of these samples, 31 had sequence information of sufficient length for additional analyses. All SAFV-2 sequences were submitted to GenBank (accession nos. JX048000–JX048030).

To determine the evolutionary history of strains of SAFV identified in persons in Denmark, we combined the VP1 sequences collected here with all others available on GenBank. We aligned sequences as described using MUSCLE software (*7*), then checked the alignments using manual calculations. We performed phylogenetic analysis using the maximum likelihood method as described in PhyML 3.0 (*8*), on the basis of the best-fit GTR+Γ nucleotide model as determined by jModelTest (*9*). Phylogenetic robustness was determined by using 1,000 bootstrap replicates.

Our phylogenetic analysis places the strains isolated in Denmark within the SAFV-2 group (Technical Appendix). These SAFV-2 strains were further subdivided into 2 strongly supported clusters: DK-A, which comprised viruses isolated during 2009–2011, demonstrating probable persistence in Denmark during this period; and DK-B, a smaller group that included viruses from United States, Germany, and the Netherlands, indicating widespread viral gene flow (Figure). The DK-B strains were identified in samples collected during 2009–2010, supporting probable sustained viral persistence within Denmark. Within DK-B, strain 115883 is phylogenetically distinct from the other DK-B viruses.

**Figure:**
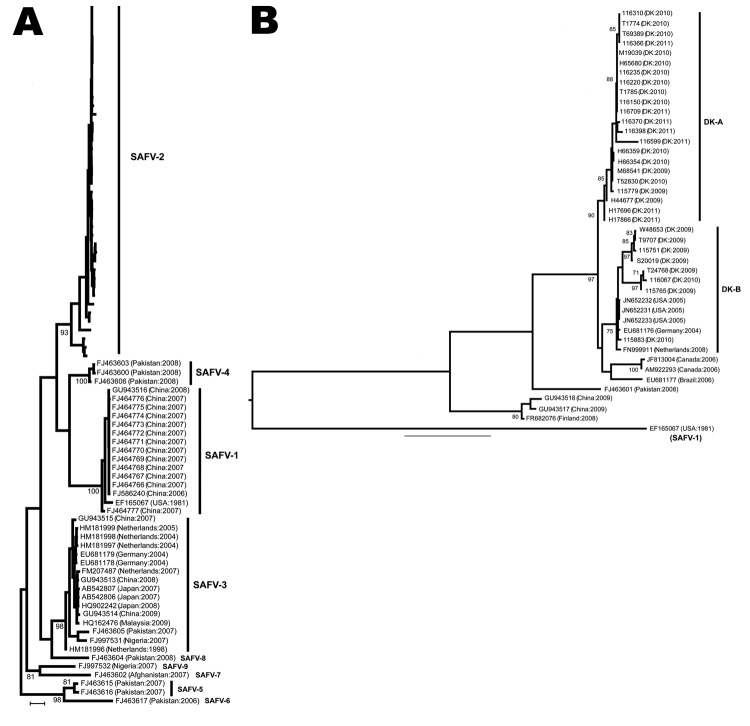
Phylogenetic analyses of Saffold viruses (SAFVs). Phylogenetic analysis of SAFV-2. Strains from Denmark are named by using the isolation numbers assigned for the study, then the country of origin and year of sampling in parentheses. The 2 subgroups (DK-A and -B) are shown. The tree is rooted by using SAFV-1 outgroup sequence accession no. EF165067. Bootstrap values >70% are shown. DK, Denmark; US, United States. Scale bars represent 0.2 nucleotide substitutions per site.

We next measured the selection pressures acting on these lineages through the mean number of nonsynonymous (d_N_) to synonymous (d_S_) nucleotide substitutions per site using the single-likelihood ancestor counting, fixed effects likelihood, and random effects likelihood methods available in the Datamonkey HyPhy package as described (*10*). The DK-A and DK-B groups differed significantly in selection pressure: DK-A, d*_N_*/d*_S_* ratio = 0.195 (95% CI 0.105–0.328); and DK-B, d_N_/d_S_ ratio = 0.033 (95% CI 0.015–0.062), which indicates stronger purifying selection on the DK-B group. Ancestral state reconstruction, performed by using Datamonkey (*10*), revealed that the ancestors of DK-A and DK-B differ only at aa 135 in VP1: Val in DK-A and Ala in DK-B. Notably, aa 135 was positively selected in DK-A (random effects likelihood: d*_N_*/d*_S_* = 3.53, Bayes factor = 50: fixed effects likelihood: d*_N_*/d*_S_* >>1; cutoff p = 0.1), with more tentative evidence for adaptation at aa 135 in DK-B: d*_N_*/d*_S_* ratio >>1 by using fixed effects likelihood (p = 0.2). The functions of aa 135 in VP1, and what it means for the fitness of SAFV, merit further consideration.

We conclude that SAFV-2 has been introduced into Denmark in 3 groups: DK-A, viral strain 115883 and strains of DK-B reported in Denmark; all have recently co-circulated in this country. We have demonstrated the entry and persistence of 3 phylogenetically distinct lineages of SAFV-2 in Denmark. That SAFV-2 can persist between years suggests that it might be common, yet underreported, in Denmark, which provides the opportunity for spread to additional localities. Increased awareness of improved laboratory protocols for SAFV detection is needed among clinicians in Denmark and neighboring countries.

Technical Appendix Epidemiologic and sequence information of Saffold virus isolated from specimens from children, Denmark, 2009–2011, and phylogenetic analyses of Saffold viruses.
