# Supplementary material for: Co-Circulation and Persistence of Genetically Distinct Saffold Viruses, Denmark
Source: Emerg Infect Dis. 2012 Oct;18(10):1694–6. doi: 10.3201/eid1810.120793 (PMC3471643; doi:10.3201/eid1810.120793)
Supplement: Technical Appendix — Epidemiologic and sequence information of Saffold virus isolated from specimens from children, Denmark, 2009–2011, and phylogenetic analyses of Saffold viruses. [file 12-0793-Techapp-s1.pdf]

Publisher: CDC; Journal: Emerging Infectious Diseases
Article Type: Other; Volume: 18; Issue: 10; Year: 2012; Article ID: 12-0793
DOI: 10.3201/eid1810.120793; TOC Head: Letters to the Editor
DOI: 10.3201/1810.120793

Co-Circulation and Persistence of Genetically Distinct Saffold Viruses, Denmark

Technical Appendix

Table. Epidemiologic and sequence information of Saffold virus isolated from specimens collected from children, Denmark, 2009–2011

Isolate name	Age/sex	Clinical symptom	Isolation date	GenBank accession no.*
T9707	16 mo/M	Ataxia	2009 Jun 6	JF693617
S20019	16 mo/F	Diarrhea	2009 Sep 11	JF693614
W48653	16 mo/M	Diarrhea	2009 Oct 6	JF693618
M68541	5 y,6 mo/M	Diarrhea	2009 Oct 1	JF693619
H66354	6 y,1 mo/F	Diarrhea	2010 Jan 1	JF693612
H66359	3 y,5 mo/M	Diarrhea	2010 Jan 1	JF693613
H44677	5 y,5 mo/M	Diarrhea	2009 Oct 14	JF693615
T52830	11 mo/M	Diarrhea	2010 Sep 27	JX048000
M19039	9 mo/F	Diarrhea	2010 Nov 14	JX048001
T69389	11 mo/M	Diarrhea	2010 Nov 27	JX048002
H17696	10 mo/F	Diarrhea	2011 Feb 9	JX048003
H17866	10 mo/F	Diarrhea	2011 Feb 9	JX048004
H65680	11 mo/F	Diarrhea	2010 Nov 11	JX048005
115751	6 mo/M	None	2009 Nov 5	JX048006
115883	6 mo/F	None	2010 Apr 20	JX048007
116142	15 mo/M	None	2010 Oct 5	JX048008
116150	6 mo/M	None	2010 Oct 19	JX048009
116177	11 mo/F	None	2010 Oct 27	JX048010
116220	6 mo/F	None	2010 Nov 11	JX048011
116235	6 mo/M	None	2010 Nov 15	JX048012
116236	16 mo/F	None	2010 Nov 14	JX048013
116310	10 mo/M	None	2010 Dec 29	JX048014

Publisher: CDC; Journal: Emerging Infectious Diseases
Article Type: Other; Volume: 18; Issue: 10; Year: 2012; Article ID: 12-0793
DOI: 10.3201/eid1810.120793; TOC Head: Letters to the Editor

Table. Epidemiologic and sequence information of Saffold virus isolated from specimens collected from children, Denmark, 2009–2011

116325	16 mo/M	None	2011 Jan 11	JX048015
116366	11 mo/M	None	2011 Jan 25	JX048016
116370	15 mo/F	None	2011 Jan 28	JX048017
116398	15 mo/M	None	2011 Feb 15	JX048018
116051	11 mo/F	None	2010 Aug 23	JX048019
116067	11 mo/F	None	2010 Sep 1	JX048020
116599	14 mo/F	None	2011 Jun 21	JX048021
116709	16 mo/F	None	2011 Oct 6	JX048022
116712	16 mo/F	None	2011 Oct 11	JX048023
115765	6 mo/F	None	2009 Nov 21	JX048024
115779	5 mo/F	None	2009 Dec 9	JX048025
116237	15 mo/M	None	2010 Nov 14	JX048026
116255	5 mo/M	None	2010 Nov 23	JX048027
116256	6 mo/F	None	2010 Nov 24	JX048028
T1774	12 mo/F	None	2010 Dec 14	JX048029
T1785	12 mo/F	None	2010 Dec 12	JX048030
T24768†	2 y,3 mo/F	Sudden death	2009 Aug 1	JF693616

*GenBank accession nos beginning with JF indicate data from previous study (*6*); all other data are from this study.
†Isolate derived from blood specimen; all other samples were from fecal specimens.
